# Main Barriers to the Introduction of a Home Haemodialysis Programme in Poland: A Review of the Challenges for Implementation and Criteria for a Successful Programme

**DOI:** 10.3390/jcm11144166

**Published:** 2022-07-18

**Authors:** Dana Kendzia, Federica Lima, Jacek Zawierucha, Ellen Busink, Christian Apel, Jacek Stanislaw Malyszko, Pawel Zebrowski, Jolanta Malyszko

**Affiliations:** 1 Fresenius Medical Care Deutschland GmbH, Else Kroener Str. 1, 61352 Bad Homburg, Germany; dana.kendzia@fmc-ag.com (D.K.); federica.lima@fmc-ag.com (F.L.); ellen.busink@fmc-ag.com (E.B.); christian.apel@fmc-ag.com (C.A.); 2 Fresenius Medical Care Poland, 60-118 Poznań, Poland; jacek.zawierucha@fmc-ag.com; 3 1st Department of Nephrology and Transplantology, Medical University of Białystok, Żurawia 14, 15-540 Białystok, Poland; jackmaly@poczta.onet.pl; 4 Department of Nephrology, Dialysis and Internal Medicine, Medical University of Warsaw, Banacha 1A, 02-097 Warsaw, Poland; zebpaw@wp.pl

**Keywords:** end-stage renal disease (ESRD), chronic kidney disease (CKD), renal replacement therapy (RRT), home haemodialysis (HHD), peritoneal dialysis (PD), in-centre haemodialysis (ICHD), large care networks, COVID-19

## Abstract

Introduction: Home dialysis in Poland is restricted to the peritoneal dialysis (PD) modality, with the majority of dialysis patients treated using in-centre haemodialysis (ICHD). Home haemodialysis (HHD) is an additional home therapy to PD and provides an attractive alternative to ICHD that combines dialysis with social distancing; eliminates transportation needs; and offers clinical, economic, and quality of life benefits. However, HHD is not currently provided in Poland. This review was performed to provide an overview of the main barriers to the introduction of a HHD programme in Poland. Main findings: The main high-level barrier to introducing HHD in Poland is the absence of specific health legislation required for clinician prescribing of HHD. Other barriers to overcome include clear definition of reimbursement, patient training and education (including infrastructure and experienced personnel), organisation of logistics, and management of complications. Partnering with a large care network for HHD represents an alternative option to payers for the provision of a new HHD service. This may reduce some of the barriers which need to be overcome when compared with the creation of a new HHD service and its supporting network due to the pre-existing infrastructure, processes, and staff of a large care network. Conclusions: Provision of HHD is not solely about the provision of home treatment, but also the organisation and definition of a range of support services that are required to deliver the service. HHD should be viewed as an additional, complementary option to existing dialysis modalities which enables choice of modality best suited to a patient’s needs.

## 1. Introduction

Kidney failure, also known as end-stage renal disease (ESRD), is the most severe stage of chronic kidney disease (CKD) resulting in an irreversible decline in kidney function [1]. The global prevalence of kidney failure is unclear but was estimated to be 5 million people in 2017 [2], with other estimates as high as 9.7 million [3].

Renal replacement therapy (RRT), either kidney transplant or dialysis, is required to sustain life [1]. While kidney transplant is regarded as the optimal type of RRT for patients, for patients who are on the transplant waiting list and for those who are ineligible to receive a transplant or have chosen not to have a transplant, dialysis is the only treatment option that can sustain life [1,4].

In-centre haemodialysis (ICHD) is the most common type of dialysis service and is performed within a hospital or other healthcare setting [5]. ICHD represents a typical way to provide medical services at a facility with specialised equipment and healthcare professionals to administer treatment and monitor patient safety. However, ICHD for ESRD patients presents some substantial challenges for health services. There is currently a global workforce shortage for kidney care, including nephrologists, nurses, and other staff, which is detrimental to the provision of care, as an effective workforce is an essential component of optimal and continuous care delivery for kidney failure management [6]. The COVID-19 pandemic has introduced additional challenges for ICHD. Patients undergoing ICHD are one of the highest risk groups among renal patients for COVID-19 due to the need for multiple centre visits per week for treatment [7]. Dialysis centres are typically clustered, close-contact environments not conducive to social distancing [8,9,10], with cases of suspected in-centre COVID-19 transmission reported [11]. Increased infection control measures have been implemented at treatment centres to reduce the risk of transmission, but even with preventative measures, outbreaks can still occur [8,12]. Furthermore, the mode of transport to and from the centre can put patients at risk, with increased risk of COVID-19 transmission reported for patients using shared transport [11,13].

Spread of COVID-19 among patients with ESRD is of particular concern. Comorbidities and epidemiological features common among these patients, such as advanced age, diabetes, hypertension, and CV disease, are prognostic factors for severe COVID-19 [7,14].

In addition to an existing shortage of qualified staff [15,16], COVID-19 may create further staff shortages due to COVID-19 illness (and recovery), the requirement to quarantine, and the need to increase capacity to allow social distancing and create isolated facilities for patients who have tested positive for COVID-19 [8,10,17].

Home dialysis is an attractive alternative to ICHD that combines dialysis with social distancing and the elimination of transportation needs [18] and also offers certain clinical [19,20,21,22,23,24,25,26,27,28], economic [29,30,31,32], and quality of life benefits over ICHD [33,34,35]. Home haemodialysis (HHD) is where dialysis treatment, either peritoneal dialysis (PD) or haemodialysis, is performed in the patients’ own home. PD uses the peritoneal cavity as a ‘natural’ filter to remove waste products contained within adjacent blood vessels; dialysis fluid is infused into the abdomen cavity and allowed to dwell for a period before draining to remove waste products [36]. HHD is conducted in the same way as ICHD; however, the patient is provided with a dialysis machine and water treatment system in their home for their dedicated use [37].

## 2. Overview of Current State of Use/Reimbursement of HHD in Poland

### Overview of HHD

Nephrologists and nurses consider frequent home or self-care dialysis to be the best long-term dialysis therapy [38], and in a survey of medical professionals, it was estimated that up to 30% of ESRD patients may be capable of performing HHD [39]. Other studies have shown that when patients receive pre-dialysis modality education, 45–60% of patients express a preference for home instead of in-centre dialysis [40]. Despite these estimates, uptake of home dialysis (PD or HHD) remains low across the majority of European countries [41].

**Figure 1:**
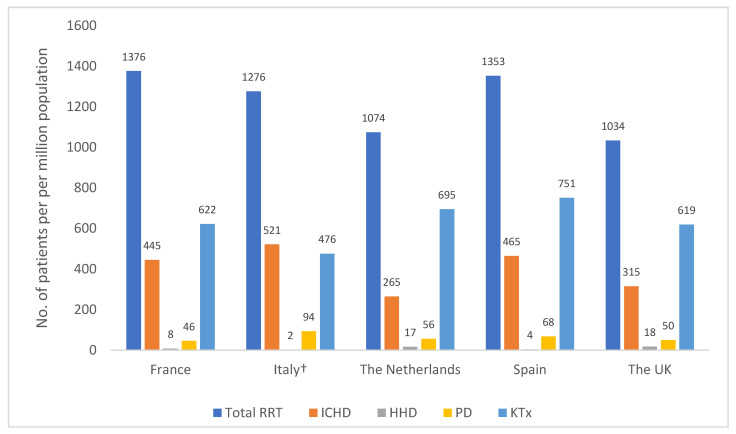
Prevalence of RRT for patients with ESRD by treatment modality (patient per million population). Figure was created using published ERA-EDTA Registry data [45]. Abbreviations: ICHD, in-centre haemodialysis; HHD, home haemodialysis; KTx, kidney transplant; PD, peritoneal dialysis; RRT, renal replacement therapy. † Representative of 8/20 healthcare regions in Italy.

HHD usage was initially prevalent in the 1960s and 1970s, with up to 59% and 32% of patients in the UK and the USA, respectively, performing HD at home due to an increasing number of patients in the face of a shortage of intra-hospital dialysis workstations [42,43]. However, despite the huge growth of patients with ESRD in the last 50 years, the percentage of patients using home dialysis has decreased since the 1970s and remains surprisingly low [43,44]; the prevalence of HHD across European countries in 2019 was 2–19 patients per million population for HHD and 45–94 patients per million population for PD. By contrast, the prevalence of patients receiving ICHD was 265–521 patients per million population (Figure 1) [41,45].

## 3. Overview of HHD and Barriers to HHD in Poland

### Overview

In Poland, almost 19,647 patients received dialysis in 2020, and the majority (95.5%) were treated with ICHD [46]. This represents a decrease from 2019, when 21,339 patients were treated, with 96.3% treated with ICHD [45]. It is estimated that approximately 25% of patients who require RRT are suitable for HHD [47]. However, currently, the 3.7% of patients who are not treated in-centre all receive PD, as HHD is not an available option [45]. Of note, there is a year-on-year trend for a decrease in the number of patients receiving PD in Poland; it is thought that this is mainly driven by patients deciding against using this treatment option due to the required patient involvement and responsibility required [48].

Although Poland has a ‘PD first’ strategy for patients, only approximately 50 public healthcare centres and a select number of private healthcare centres are able to perform PD treatment [49]. Patients receive training at these centres, and the necessary equipment and materials are delivered by the supplier to the patient’s home at initiation with regular intervals scheduled (every 2–4 weeks) to maintain treatment. The patient’s physician is responsible for prescribing the PD treatment, and the patient contacts the physician or centre if any complications occur as a result of therapy. The patient is required to regularly visit the centre for monitoring every 4–6 weeks.

There are no national recommendations or guidance for PD or HHD in Poland. However, a clinic must have a license to provide PD and must meet certain conditions for dialysis provision (e.g., infrastructure, personnel requirements, training) described by National Fund documents. This places limitations on the number of clinics that are able to offer this service. The National Health Fund currently provides a fixed reimbursement rate to the centre for daily treatment regardless of PD modality (i.e., automated PD or continuous ambulatory PD), and no costs are passed through to the patient.

## 4. Barriers to HHD

The use of the patient’s home is one of the main requirements for the provision of HHD. In Poland, due to legal restrictions (either central or local government), it is not possible to perform haemodialysis at the patient’s home [37]. Additionally, also due to the absence of specific legislation, it is not possible to obtain insurance to perform HHD or for clinicians to prescribe the treatment. In theory, a university centre can perform experimental therapies, which could cover a program that delivers HHD. However, this would be both logistically demanding and expensive to set up, as well as unclear as to who would bear the cost of any claims from the patient. A potential step towards the establishment of HHD in Poland could be consultation between scientific societies and the Ministry of Health to design and implement a pilot study for HHD. If the pilot study proves beneficial to patients and payers, then subsequent steps could be taken, such as the provision of a legal framework, in order to establish HHD as a treatment choice for patients in Poland. Specific health legislation to allow HHD should define who is responsible for patient safety in the home and how the treatment will be supervised. Additionally, the conditions of reimbursement (staff, equipment, lab tests, modality, etc.) must be defined.

To introduce a new medical service such as HHD in Poland, several steps are required. Firstly, the National Consultant, Scientific Society or Patients Association makes an application to the Ministry of Health, on the basis of a literature review and clinical analysis, for a new medical intervention. Next, a HTA is required to assess the new medical intervention, which covers topics such as costs, budget impact analyses, and price calculations. Lastly, guidelines are required for the new intervention (i.e., staff requirements, lab tests, dialysis frequency, training, equipment, etc.).

In addition to legal issues, there are further downstream barriers to overcome. For example, there may be technical issues for installation of HHD, as adaptations to a patient’s home are required (modifications to electricity supply, provision of a purified water supply) and the dwelling type (e.g., apartments, first floor houses) may not be amenable to these modifications.

Clear rules are required for costs, including who pays for the installation of HHD in the patients’ home; materials required; patient training; and other services, such as delivery of materials and waste collection. Additionally, logistics required for service delivery must be defined and established, such as installation of equipment, regular delivery of materials, and collection of waste. Infrastructure and experienced personnel are also essential for the training and monitoring of patients on HHD. Clinicians and patients may require convincing that any complications that arise during HHD can be managed effectively, as the support provided by the renal ward during ICHD is not immediately available with HHD. Digital support platforms, such as telemonitoring, may assist with overcoming such barriers to HHD. A study which investigated remote monitoring of patients’ HHD sessions found that it helped lower the risk of treatment discontinuation and also increased the proportion of patients who successfully completed HHD training [50]. Although remote monitoring is currently not widely used in HHD, its implementation has the potential to address fears regarding responsibility for patient safety at home, from a legal, provider, clinician, and patient perspective. However, further research is required to determine the effectiveness of telemonitoring for patient safety in a HHD setting.

Furthermore, despite all these barriers, the existing provision of PD in Poland (including experiences of the establishment and maintenance of service) may be leveraged to assist the introduction of HHD (e.g., regular monitoring of patients in centre, legal framework required for the provision of treatment at home).

## 5. Additional Barriers to HHD Implementation (i.e., Not Country Specific)

Aside from the high-level organisational, reimbursement, and legal barriers that must be addressed to set up a HHD programme in Poland, there are a number of additional barriers that are common to the establishment and uptake of HHD, regardless of the country or region.

### 5.1. Patient Level Barriers

For patients, a lack of motivation to perform HHD [51,52] and fears regarding the dialysis process (e.g., operating the machine, self-cannulation, risk of catastrophic events) [33,51,52,53,54] are two main factors. There is a high drop-out rate from training and also from treatment (as a result of home suitability, ability to cope with the burden of HHD, worsening medical conditions, inadequate support, etc.) [55]. Where families and carers are required to assist with activities such as responsibility of assisting patients with treatment, monitoring adherence, advocating for patients, and attending appointments [35,56], limited capacity and time availability for these activities may preclude HHD. Other patient-level barriers to HHD include a lack of satisfactory explanation of various techniques [57], family burden [58], fears specifically regarding change [57], failure to perform the treatment adequately [57,58], or of social isolation [33,57]. It must be noted that many of these fears are overstated and may be addressed by providing patients with education and training that allays their fears and provides confidence in their ability to conduct HHD [59]. Consequently, education and training are critical component for driving HHD uptake.

### 5.2. Education and Training

Absence of or limited education and training for HHD, for both patients and clinicians, is a major barrier, representing an area which requires improvement to drive HHD uptake. Patients require a sufficient level of health literacy and provision of training by experienced staff in order to conduct HHD safely, effectively, and with confidence. Lack of adequate patient and caregiver education is a factor that has led to low rates of HHD uptake, resulting in patients receiving ICHD [54].

On the basis of a survey of patients with ESRD in Europe, almost 40% were not provided information or education about alternative dialysis treatment options to their current modality [60]. Potential reasons for inadequate patient education include a lack of familiarity with home dialysis and candidacy bias among treating physicians and nurses [3]. Some hospitals and clinicians may take a paternalistic approach to treatment and assume that only a limited number of patients can effectively manage their treatment at home, resulting in clinicians prescribing ICHD to patients that are eligible for home dialysis if prepared correctly (i.e., with education on dialysis modalities) [61]. This approach may be driven by perception of low healthcare literacy among some patients, which may be common among patients undergoing dialysis [62].

Clinician education is also important for HHD uptake. Evidence indicates that nephrologists may lack exposure to home dialysis modalities, with many believing that HHD is too complicated and burdensome for the majority of patients with kidney failure [47,54,63]. Clinician inertia may also be a challenge, which may arise from unfamiliarity, as many physicians receive training that does not require experience with HHD [64]. This may result in a lack of knowledge among physicians on how to establish HHD programs and how to adequately manage HHD training and care [64]. Furthermore, the lack of exposure of clinicians to HHD may hinder the development of clinical advocates or ‘clinical champions’ for home dialysis, which have been shown to have a beneficial influence on HHD uptake [65]. There are educational programs that train clinicians and administrators to develop new home dialysis programs; however, these programs are limited in number [66].

Another issue which hinders patient education is the situation termed ‘crashing into dialysis’, where patients are referred late to the clinic and are in urgent need of dialysis [67]. Because of the urgency, these patients begin dialysis in hospital without the requisite time for education about dialysis modality options, and the opportunity to begin on home dialysis is missed [68]. For a country where HHD is newly introduced, education should be a major consideration as it is an important component for promoting HHD uptake. Despite this, education remains an issue in countries with the existing legal framework required to provide HHD [54,63,68].

### 5.3. Reimbursement and Costs

In addition to the reimbursement challenges previously discussed for Poland, there are also additional considerations. From a payer perspective, when HHD reimbursement is established, a low level of reimbursement, reimbursement schemes which are not sufficiently flexible to cover the costs of more frequent HHD prescriptions [54,69,70], and hidden costs that exist for HHD which are not reflected in the reimbursement rate can also inhibit uptake [71]. With regard to hidden costs, there is poor clarity around the aggregated costs for single items that are required to produce dialysis equipment for both PD and HHD and the labour costs involved in delivering HHD [31]. Consequently, this makes it difficult to determine an accurate reimbursement level [3].

From a provider perspective, the infrastructure and resources required for training patients represents a major barrier to HHD use due to the substantial upfront expenditure required for facilities and necessary staff to train patients [72,73]. Additionally, providers normally expect to recover their initial investment over time through provision of services. However, there is a risk that patients drop out of the HHD program (e.g., kidney transplant or develop a preference for ICHD), with providers unable to recover the costs for these patients (i.e., training).

Increased patient expenditure related to treatment may also represent an impediment to HHD, as depending on the reimbursement level, patients (or their families) may have to cover certain costs of treatment (i.e., co-payment for drugs or consultation, which often increase as CKD progresses) [3,54,73,74].

## 6. Clinical, Economic, and Holistic Benefits of HHD

HHD offers clinical benefits over ICHD. HHD typically allows a more flexible treatment schedule than ICHD, which can be defined on the basis of the patient’s medical need and lifestyle [19]; an example of selected treatment schedules is presented in Table 1.

Evidence suggests other benefits of HHD, including a lower mortality rate [20,21,22,23,24,25] and a quicker recovery time compared with ICHD [26]. HHD may also be associated with reduction in antihypertensive medication [79]. Additionally, HHD may be linked to fewer adverse outcomes than ICHD [27,28]. Although the evidence is limited, studies have reported that HHD is associated with a lower rate of annual hospitalisation, CV-related admissions, and annual hospital length of stay compared with ICHD [27,28]. A key benefit of HHD during the COVID-19 pandemic is that patients can receive their treatment at home, reducing face-to-face contact with hospital staff, transport drivers, and other patients [18,80], which can reduce the risk of contracting COVID-19 [8]. Consequently, studies have shown that patients undergoing home dialysis are almost 50% less likely to develop COVID-19 versus those undergoing ICHD [81].

HHD may also offer economic benefits over ICHD. Although HHD may be associated with higher upfront costs than ICHD due to the need for home set up and training, these may be offset in the long-term, as ICHD incurs greater costs due to staffing, facility costs, and patient transport [82,83], ultimately resulting in lower overall costs for HHD [29,30,31,32]. However, these studies did not assess additional costs of HHD, such as transportation of equipment/consumables, waste management, and call centres. Consequently, there is a need for further research to comprehensively assess the cost benefit of HHD compared with ICHD. On the basis of current evidence, the main economic benefits of HHD fall outside the reimbursement bundle (i.e., the main economic benefits are for healthcare systems rather than payers), which makes HHD an attractive option for healthcare systems (i.e., reduced use of in-hospital resources, etc.). However, the benefits of HHD to healthcare systems must not be viewed as a justification by payers to reduce reimbursement levels. Given that PD is currently the only reimbursed home dialysis treatment in Poland, it should be expected that similar reimbursement is provided for HHD if it was established.

From a patient QoL perspective, patients and caregivers describe HHD as offering improved freedom, the ability to regain their social life, work full time, take care of children, and spend time with their family [33,34,35]. These benefits are supported by the flexibility of dialysing at home and time saved (i.e., no travelling time to a dialysis centre required) [84]. Additionally, although the evidence is mixed, HHD may offer environmental benefits, with evidence indicating that HHD is associated with a reduction in CO_2_ emissions per patient annually compared to hospital-based HD [85].

## 7. Requirements to Set up a Successful Program

There are several requirements for a successful HHD program, assuming all high-level legal and reimbursements issues have been addressed. Firstly, a multidisciplinary team consisting of nephrologists, nurses, dieticians, social workers, and other HCPs is required [86]. In addition, medical infrastructure will need to be created (or adapted from existing facilities) to provide an adequate location where patients can be trained to perform HHD under supervision before they can dialyse at home.

Patient suitability should be determined on an individual basis and predominantly driven by patient preference and medical suitability (Table 2) [87].

HHD requires specific organisation and infrastructure in the patients’ home before it can be set up [37]. From an infrastructural perspective, the building must be in good condition (i.e., not affected by dampness, mould, or excessive environmental pollution), availability of appropriate electricity supply (i.e., stable electricity supply), and sufficient water supply and methods of communication (i.e., telephone, internet) [37].

Patient training is a critically important requirement before initiating HHD. Patients are required to undergo an intensive training programme to facilitate successful management of their own treatment, with training programmes ranging from several days to 3 months [19,76,92]. Training aims to provide patients with sufficient information to be able to dialyse at home, to help them overcome any barriers and fears associated with HHD (such as those previously discussed), and to successfully manage other elements of their ESRD such as diet [88]. While some patients may only be able to perform HHD with the help of a nurse, most patients perform the treatments themselves, either alone or with the assistance of a dialysis helper (usually a family member or friend) [92,93]; provision of training is also required for the nominated dialysis helper.

The dialysis provider is responsible for installing the necessary equipment, including the dialysis machine and a mobile water treatment system, and making any necessary adaptations required to the patient’s home. The provision of HHD support services is also important. Patients receive regular deliveries of consumables such as dialysers, needles, machine lines, and other consumables (e.g., bandages, medical tape, cleaning materials) which they are required to store in their home [19,94], in addition to regular collection of medical waste [37]. The location of the dialysis machine is important; the room chosen for HHD should be functional and conducive to safe and convenient HHD [37]. For example, patients receiving nHHD will need to perform dialysis in their bedroom while patients performing HHD during daytime hours may wish to perform their dialysis in another room [37]. In addition to training, ongoing support for patients is also required, with 24 h access to a technical support team and regular check-up appointments with their care team [95].

## 8. Example of Successful Program Establishment in Turkey

Where a HHD programme does not yet exist, there are several key components required for its set up and growth. To highlight the key components of the successful establishment of a large care network including HHD, the details of a case study from Turkey are described.

Initially, the service needs to be introduced as a pilot scheme with low patient numbers and grown gradually over time (years) to ensure that adequate infrastructure, staff, and support services (i.e., the supply of medical items and waste disposal) are available to provide for more patients. Crucial to the development was the identification of a ‘HHD champion’ (i.e., a clinical advocate) that initially took charge of the HHD pilot scheme. The clinical advocacy in the initial stages is also key to raising awareness about the benefits of HHD to many stakeholders, including the public, patients, clinicians, and payers. Evidence from other countries indicates that clinical champions can facilitate uptake of HHD [65].

**Figure 2:**
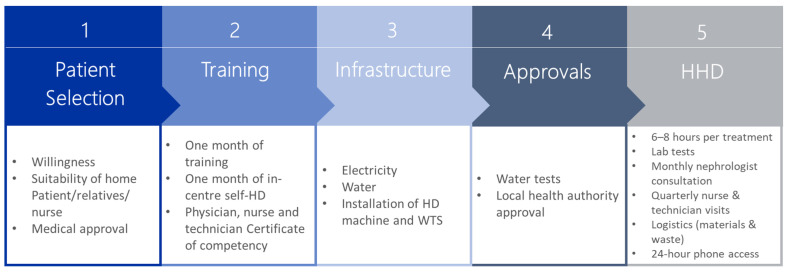
Steps in the HHD pathway. Abbreviations: HD, haemodialysis; HHD, home haemodialysis; WTS, water treatment system.

After the infrastructure, staff, and necessary support services are in place, selection of eligible patients is required, followed by training to dialyse at home. Training is delivered at a dialysis centre where the patient performed self-HD under close supervision. The training included input from physicians, nurses, and technicians. Infrastructure in the patients’ home must be established, i.e., availability of electricity and water supply and the subsequent installation of the HD machine and water treatment system. Approval from the local health authority is required, and when the service is up and running, the support services required to sustain the HHD and ensure the patient is receiving the optimal treatment must be maintained. These services include logistical services (i.e., delivery of consumables required for dialysis and the removal of waste generated) and clinical services (i.e., monitoring of the patient using lab tests, 24 h phone access to support staff, and consultations with support staff). An overview of the steps in setting up the HHD pathway is provided in Figure 2.

## 9. Partnering with a Large Care Network May Also Facilitate a Successful HHD Programme

As previously mentioned, a substantial investment is required for the infrastructure, staff, and support services required to establish a new HHD program. In addition to investment, there are other issues to contend with, such as staff shortages, a lack of experience in developing such a care system, and the availability of experienced clinicians familiar with educating and training patients for HHD [6,64,66].

An alternative option for payers is to rely on large care networks to provide a HHD service. A large care network is a group of care providers specialised in one or multidisciplinary disease areas that has dedicated infrastructure, staff, and processes to facilitate the provision of patient-centred, evidence-based high-quality care [96,97,98]. Across a variety of chronic diseases, the integrated care delivered by care networks has several beneficial outcomes, including reduced mortality, reduced hospital admissions and readmissions, improved quality of life, and adherence to treatment guidelines [98]. In addition to these benefits, large care networks for HHD also offer a continuous supply of services and staff alongside substantial experience of service delivery. Large care networks which deliver ICHD can facilitate the introduction of an HHD care network as existing infrastructure, and networks can be modified to provide support for HHD. Importantly, these large care networks have vast experience in delivering services, providing a continuous availability of experienced staff, dedicated infrastructure, and support services and supplies to support patients [99]. Even if a specific large care network is not yet established in a country where partnership is sought, best practice exchanges are possible. For example, this may include sending clinicians to other countries where a care network has been established to observe, train, and gain experience which can be used to assist service development in their home country. The main pillars of the Chronic Care Model (CCM) are presented in Table 3; many of these pillars address current barriers to HHD, including training and education of both staff and patients [96,100,101].

## 10. Conclusions

Despite the challenges and barriers to introducing HHD in Poland, there are clear benefits of HHD which make it a worthwhile investment. From a patient perspective, dialysing at home provides flexibility and saves time [84], as well as improving QoL [33,34,35]. Evidence indicates clinical benefits such as greater patient survival [20,21,22,23,24,25], reduced hospitalisations [27,28], and adverse outcomes compared with ICHD [27,28]. From an economic perspective, HHD may be associated with higher upfront costs than ICHD, but these may be offset in the long term due to lower costs for staffing, facilities, and patient transport [29,30,31,32,82,83]. ICHD remains the mainstay of treatment in most developed countries despite all the potential benefits of HHD, reflecting the historical allocation of healthcare resources, and is embedded in physician training and perception of HD delivery [84]. Providing HHD is not solely about the provision of equipment and materials to facilitate home treatment, but also a holistic service programme which organises and defines a range of support services that are required to deliver the service. To facilitate the introduction of HHD in Poland, identification and recognition of barriers is the first step. Establishment of HHD will require changes that are affected from the top down (primarily the creation of specific healthcare legislation, but also other regulations, reimbursement incentives, and organisational changes), and bottom-up efforts (patient awareness of treatment options and motivation to perform HHD) will also be required. The introduction of HHD should not be viewed as a replacement for other dialysis modalities, but rather to complement existing dialysis modalities and provide additional options in the management pathway to provide patients with the modality that is best suited to their individual needs. In Poland, these forms of therapy can be improved and extended to more patients, similarly to many other countries.

The review was based on the search in all available databases using mesh terms ‘home haemodialysis’, ‘renal replacement therapy’, ‘in-centre haemodialysis’, ‘peritoneal dialysis’, ‘reimbursement’, ‘cost quality of life’, etc. All possible source information, in particular for Poland, were retrieved from available sources such as the ERA-EDTA registry, data from National and regional consultants on the current renal replacement, and reimbursement form National Health Fund. Contract information. 2021 [45,46,49].

## Figures and Tables

**Table 1:** Example of selected HHD treatment schedules.

Conventional HHD (cHHD)	Treatment lasts approximately 4 h and is performed 3 times per week; this is the typical treatment schedule which is offered to patients undergoing ICHD [75,76,77]
Short daily HHD (sdHHD)	Treatments are performed 2–3 h per day for 5–7 days per week [75,76,77]
Nocturnal HHD (nHHD)	Treatments are performed for 6–10 h overnight while the patient is asleep for 3–7 nights per week [75,76,77]
Every other day HHD	Treatments are performed during the daytime, every other day [78]; the length of the treatment may vary but is usually 4 h [75]

Note: This list is not exhaustive, other treatment schedules are available.

**Table 2:** Selected patient eligibility criteria for HHD.

**Suitable patient characteristics for HHD**
Physically and mentally capable and willing to learn and manage their dialysis [88]. In some instances, a committed family member or friend who is willing to train and support them with their dialysis treatments [89]. A suitable home environment to house machinery and consumables, as well as the ability to make modifications if required (for example to plumbing and electricity supply) [19,37,88,90]. Those who wish to continue with work or education [88]. Women who are pregnant or wish to conceive [88,91]. Patients with a range of medical conditions including severe uncontrolled sleep apnoea, persistent hyperphosphatemia, right heart failure, uncontrolled ascites, refractory volume overload, and difficulty in controlling hypertension [88]. Patients who experience issues with conventional HD such as excessive recovery time, inadequate control of uremic symptoms, and symptoms such as hypotension cramps or nausea [88].
**Patient characteristics which may be considered contraindications for HHD [88]**
Certain medical conditions (e.g., uncontrolled arrhythmia, seizure disorders, conditions causing abrupt loss of consciousness). Certain mental health conditions (uncontrolled psychosis or anxiety, drug use, alcohol abuse). Contraindications for anticoagulation use.

Abbreviations: HD, haemodialysis; HHD, home haemodialysis.

**Table 3:** The main pillars of care networks and associated activities.

Main Pillars	Associated Activities
1. Health system or health organisation	Best practice exchange between clinics [99] Continuous availability of supplies, services, and staff [99] Partnerships in health system—collaboration, co-ordination, and integration of services [96,98]
2. Clinical information systems	Continuous monitoring of patient data [102] Allows for setting of key performance indicators and benchmarking [102]
3. Decision support	Implementation of evidence-based guidelines [103] Use of predictive analytics [104] Continuous education and training of staff [96,103]
4. Delivery system design	Standardisation of treatment and workflows [105]
5. Self-management support	Systematic implementation of patient education [96,105,106]

## Data Availability

The data presented in this study are available on request from the corresponding author.
